# Development and Validation of a Predictive Model of Pain Modulation Profile to Guide Chronic Pain Treatment: A Study Protocol

**DOI:** 10.3389/fpain.2021.606422

**Published:** 2021-02-15

**Authors:** Matthieu Vincenot, Alexia Coulombe-Lévêque, Monica Sean, Félix Camirand Lemyre, Louis Gendron, Serge Marchand, Guillaume Léonard

**Affiliations:** ^1^Research Center on Aging, Université de Sherbrooke, Sherbrooke, QC, Canada; ^2^Faculty of Medicine and Health Sciences, Université de Sherbrooke, Sherbrooke, QC, Canada; ^3^Department of Pharmacology-Physiology, Faculty of Medicine and Health Sciences, Université de Sherbrooke, Sherbrooke, QC, Canada; ^4^Centre de Recherche du Centre Hospitaliser Universitaire de Sherbrooke, Sherbrooke, QC, Canada; ^5^Département de Mathématiques, Faculté des Sciences, Université de Sherbrooke, Sherbrooke, QC, Canada; ^6^Faculty of Medicine and Health Sciences, School of Rehabilitation, Université de Sherbrooke, Sherbrooke, QC, Canada

**Keywords:** chronic pain, temporal summation, conditional pain modulation, pain modulation profile, catecholamines, clinical feasibility

## Abstract

**Introduction:** Quantitative sensory testing is frequently used in research to assess endogenous pain modulation mechanisms, such as Temporal Summation (TS) and Conditioned Pain Modulation (CPM), reflecting excitatory and inhibitory mechanisms, respectively. Numerous studies found that a dysregulation of these mechanisms is associated with chronic pain conditions. In turn, such a patient's “profile” (increased TS and/or weakened CPM) could be used to recommend different pharmacological treatments. However, the procedure to evaluate these mechanisms is time-consuming and requires expensive equipment that is not available in the clinical setting. In this study, we aim to identify psychological, physiological and socio-demographic markers that could serve as proxies to allow healthcare professionals to identify these pain phenotypes in clinic, and consequently optimize pharmacological treatments.

**Method:** We aim to recruit a healthy participant cohort (*n* = 360) and a chronic pain patient cohort (*n* = 108). Independent variables will include psychological questionnaires, pain measurements, physiological measures and sociodemographic characteristics. Dependent variables will include TS and CPM, which will be measured using quantitative sensory testing in a single session. We will evaluate one prediction model and two validation models (for healthy and chronic pain participants) using multiple regression analysis between TS/CPM and our independent variables. The significance thresholds will be set at *p* = 0.05, respectively.

**Perspectives:** This study will allow us to develop a predictive model to compute the pain modulation profile of individual patients based on their biopsychosocial characteristics. The development of the predictive model is the first step toward the overarching goal of providing clinicians with a set of quick and cheap tests, easily applicable in clinical practice to orient pharmacological treatments.

## Introduction

Chronic pain, which affects one in five people worldwide (1), has been defined as pain that persists beyond the usual healing time (2). However, merely relying on this temporal aspect is not enough to account for the complexity of chronic pain vs. acute pain. As such, the notion of inadequate response to treatment and significant, lasting alterations of functional and psychosocial status have increasingly been recognized as an integral part of this condition (3). Several factors have been associated with chronic pain, including physical limitations (4, 5), cognitive impairments (6), and psychological conditions, such as anxiety and depression (7).

Dysregulations of endogenous pain modulating systems are also thought to be involved in the development of chronic pain. These dysregulations include increases in pain facilitation [which amplify our body's alertness (8)], and decreases in pain inhibition [which triggers diffuse hypoalgesia (9)]. These endogenous pain modulation mechanisms can be measured using quantitative sensory testing based on Temporal Summation (TS) and Conditioned Pain Modulation (CPM), respectively. Both of these testing paradigms have high test-retest reliability (10–12).

TS is the psychophysical correlate of central sensitization, which is defined as an increased responsiveness of second order neurons (located in the spinal cord) to afferent C-fibers (13) resulting in a heightened pain perception (14). Increased TS is thought to be involved in various chronic pain conditions, such as fibromyalgia (14, 15), chronic low back pain (16), and painful knee osteoarthritis (17). CPM, on the other hand, refers to the phenomenon wherein a noxious stimulation results in widespread hypoalgesia (18), akin to the diffuse noxious inhibitory control involving a spino-bulbo-spinal loop studied in animal models (9, 19). Weakened CPM is found in chronic pain conditions, such as chronic tension headaches (20), provoked vestibulodynia (21), atypical trigeminal neuralgia (22), temporomandibular disorders (23), fibromyalgia (24), and irritable bowel syndrome (23).

TS and CPM measures can be used to identify individuals at risk of developing chronic pain (25). For example, the amplitude of pre-operative TS is correlated with the intensity of post-operative pain (26), while a placebo-controlled study has found that weaker CPM is associated with a better response to duloxetine (a serotonin-noradrenalin reuptake inhibitor) in patients suffering from neuropathic pain (27). This line of research has led authors to suggest that it is the presence of specific deficits in endogenous pain modulation mechanisms, rather than the type of chronic pain, that best predicts response to treatment (28, 29). Indeed, according to Yarnitsky and colleagues, each individual can be placed along a spectrum ranging from an *anti*nociceptive (normal TS/effective CPM) to a *pro*nociceptive (increased TS/weak CPM) profile, and as such should be more receptive to an individually tailored treatment that specifically counteracts their deficits (25).

The evaluation of these endogenous mechanisms could therefore prove useful in the clinic, by providing healthcare professionals with valuable information regarding the pain modulation profile of patients to help them choose the most appropriate pharmacological approach. Unfortunately, to the best of our knowledge, no study has attempted to estimate the amplitude of TS and CPM using readily accessible measures. Protocols currently used to evaluate endogenous pain modulation mechanisms are time consuming and require expensive, complex equipment that would not be practical for daily clinical practice. As such, an alternative approach, which would allow clinicians to easily estimate their patient's CPM and TS using readily available tests, would be highly relevant to clinical practice.

Despite the lack of predictive models using TS and CPM as outcome, numerous association studies suggest that certain factors influence endogenous mechanisms. TS and CPM have long been known to be affected by age and sex (30–38), but more recently they have also been linked to a number of other psychophysiological variables. For instance, TS is consistently associated with pain catastrophizing (39–42), whereas CPM is modulated by both catastrophizing (43, 44) and expectations (45); additionally, a wide array of other psychological factors have been related to pain mechanisms, albeit in a method-dependent manner [for a review, see (46)]. Certain physiological factors, such as blood pressure, also seem to correlate with TS/CPM (47, 48) response, whereas others, such as activation of endocannabinoid and opioid systems, are known to directly contribute to pain inhibition (49–51). Moreover, a lower plasma concentration of dopamine (52) and catecholamines (norepinephrine and serotonin) (53) is associated with deficient CPM in chronic pain patients and, interestingly, it seems that this low plasma concentration is attributable to genetic polymorphisms, which suggests that CPM deficiency could in fact have a genetic correlate (54).

Different approaches have been developed (based on similar theoretical frameworks) to assess endogenous mechanisms. For TS, constant (47, 53–55) and pulsed (15, 31, 56, 57) nociceptive stimulations are commonly used, with a large variety of stimuli (58). For CPM, protocols include parallel (27, 34, 59, 60) or sequential (35, 38, 61–63) paradigms. In this context, the heterogeneity of the aforementioned studies and approaches makes it difficult to draw any meaningful generalization (64). Moreover, these factors have yet to be systematically evaluated within a single study.

The first objective of this study is to develop and validate a predictive model to estimate the amplitude of TS and CPM. Our second objective is to estimate reference values of these endogenous excitatory and inhibitory modulation mechanisms of pain in a healthy population.

## Methods

### Participants

Our participants will take part in a single experimental session, taking place at the Centre de Recherche du CHUS (Sherbrooke, Quebec, Canada). Eligibility criteria (56) are listed in Table 1. We will ask that participants refrain from consuming tobacco (65, 66), alcohol (67, 68), and stimulants (69) (caffeine, theine, etc.) in the 24 h preceding the experimental visit, and that they skip their last dose of pain medication (acetaminophen, non-steroidal anti-inflammatory drugs, and narcotics), such that they are not under the influence of an analgesic agent during the session. Recreational substances are also prohibited up to 7 days before the testing. Participants who fail to adhere to these instructions will be asked to reschedule their session. Participants will be recruited using volunteer registers; Facebook advertisement, posters, and leaflets; those interested will be invited to contact the team and to complete screening questionnaires to confirm their eligibility.

**Table 1:** Eligibility criteria.

	**Chronic pain patients**	**Healthy Participants**
Inclusion criteria	Able to understand instructions	
	18–79 years of age	
	Chronic Pain condition (>6 months)	Free from chronic pain condition
Exclusion criteria	Chronic headache	
	Pregnancy, or post-partum (<1 year) status	
	Active cancer or cancer-related pain	
	Cardiac or vascular diseases	
	History of psychotic or neurocognitive disorder	
	Regular use of recreational substances	
	Acute or chronic arm injury, including impaired sensitivity	

### Independent Variables

#### Psychological Distress

A number of psychological conditions are comorbidities of chronic pain (7). For this reason, different psychological variables will be evaluated.

##### Anxiety and Depression

The *Hospital Anxiety and Depression Scale* is an instrument used to screen for anxiety and depressive disorders. It comprises 14 items (seven each for anxiety and depression) rated from 0 to 3, for a maximum score of 21. Subjects are classified as presenting no symptoms (≤7), mild symptoms (8–10), or moderate to severe symptoms (≥11) for both disorders. The psychometric properties of this questionnaire are qualified as excellent (70).

##### Catastrophizing

The *Pain Catastrophizing Scale* (71) is a 13-item questionnaire representing the three components of pain catastrophizing: rumination, magnification, and helplessness. A score above 30/52 indicates a clinically significant level of catastrophizing. This questionnaire has good psychometric properties (72).

#### Pain Measurements

##### Pain Intensity

The *Brief Pain Inventory (BPI)* (73) will be used to measure pain intensity and interference with daily life. The BPI is a generic, self-administered pain questionnaire for chronic pain conditions. The psychometric qualities of this test are excellent (74–76).

##### Pain Type

The *Douleur Neuropathique 4* (77) is a diagnostic tool used to identify neuropathic pain. It assesses pain symptoms (type of pain and presence of dysesthesia, out of seven) and signs (physical examination, out of three). A score ≥ 4/10 suggests a neuropathic component. This tool is regularly used in research, and has excellent psychometric properties (77).

##### General Clinical Pain

A pain diary will be used to measure daily clinical pain. Participants with chronic pain will be asked to indicate the highest, average and lowest level of pain they feel on seven occasions (3 days prior to the visit, the day of the visit and the 3 days following the visit).

##### Somatization

The *Somatization* subscale of the *Symptoms Checklist 90* (78, 79) will be used to assess somatization in our participants. The subscale comprises 12 items, each rated on a 5-point scale. Psychometric properties of this subscale are excellent (79).

##### Mechanical Pain Threshold

A digital algometer will be used to assess the mechanical pain threshold (i.e., the point at which a mechanical pressure starts to elicit pain). The algometer will be placed on the upper trapezius muscle, and the pressure will be gradually increased until the participant verbally reports pain. Four trials will be conducted—two on each side—and the average pressure required to reach the mechanical pain threshold will be recorded. This test has good to excellent reliability and validity (80–82).

##### Pain Persistence

A digital algometer will be used to assess pain persistence. Following the mechanical pain trials described above, the algometer will be applied on the right upper trapezius muscle, and pressure will be increased until participants verbally report a pain intensity of 5/10 (numerical rating scale). This pressure will be maintained for 20 s (Tonic Mechanical Pain test). Immediately after the algometer is removed, participants will be asked to rate the intensity of their lingering pain sensation (if any), again using the numerical rating scale. Pain scores will subsequently be collected every 2 min, until participants report two consecutive scores of 0/10.

#### Physiological Measurements

##### Blood Pressure

The cardiovascular system and the descending inhibitory pathways of pain appear to be closely related (48, 75). Blood pressure will be measured three times (at rest, before, and after the conditioning stimulus—see below) using a digital sphygmomanometer.

##### Blood Concentration

**Catecholamines** (53) (adrenaline, noradrenaline, dopamine, serotonin, and their metabolites), **Endocannabinoids** (anandamide, N-olcoylethanilamine, palmitoylethanolamine), and **Peptides** (83, 84) (Leu-Enképhaline, B-Endorphin, Dynorphine A, Substance P). Blood samples will be collected in K2-EDTA tubes and will be placed directly on ice. After centrifugation (4°C, 1,500 g, 15 min), samples will be directly stored at −80°C before analysis. The different analytes will be separated and analyzed by liquid chromatography coupled to a tandem mass spectrometer. The analysis will be carried out at the bioanalysis platform of the Sherbrooke Pharmacology Institute.

### Dependent Variables

#### CPM and TS Amplitude

The amplitude of CPM and TS will be assessed within a single experimental procedure, developed by Tousignant-Laflamme (85) and in accordance with Yarnitsky (18, 86). This procedure consists of a Test Stimulus applied before and after a Conditioning Stimulus (CS); the amplitude of TS is measured as the changes in pain levels observed throughout the first administration of the Test Stimulus, and the amplitude of CPM is measured as the difference in pain levels elicited by the Test Stimulus before and after the CS. This method is commonly used to measure these mechanisms (35, 55, 87, 88).

#### Pain Perception

Pain perception will be assessed using a Computerized Visual Analog Scale (CoVAS). This tool, highly validated and commonly used in research (89–91), consists of a slider placed along a visual analog scale (left end: 0, or no pain; right end: 100, or worst pain imaginable), linked to a computer. Participants move the slider along the scale according to their pain levels, such that their perceived pain intensity is continuously recorded by the computer.

### Procedure

**Figure 1:**
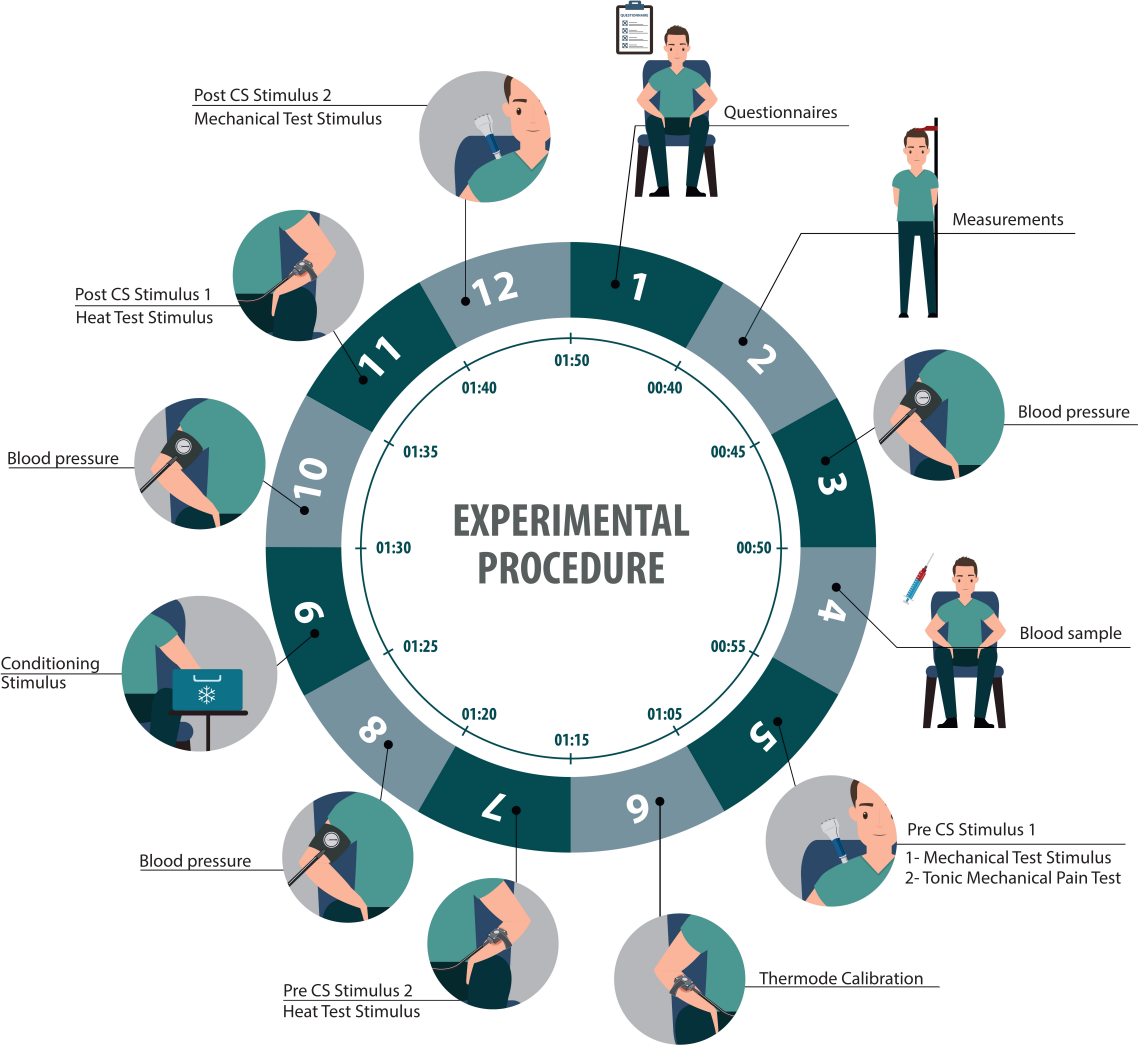
Illustration representing the sequence of the experimental procedure.

At the beginning of the session, we will reassess participants' eligibility, and we will collect their informed consent. We will also collect data relating to our independent variables, using a series of questionnaires, physiological measures, and a blood sample. All questionnaires will be provided in each participant's preferred language (English or French). We will then assess the amplitude of their CPM and TS using quantitative sensory testing (Figure 1):

#### Thermode Calibration

Pre-tests will be conducted using an experimental thermode (9 cm^2^, TSA II), at a temperature increasing from 32° to 51°C at a rate of 0.3°C/s. Participants will be asked to identify the point at which heat becomes painful (heat pain threshold) and the point at which it becomes intolerably painful (heat pain tolerance).

One pre-tests will be conducted on the left hand of each participant, so they become familiar with the thermode and CoVAS. Three more pre-tests will be conducted on the anterior side of the left forearm, to identify the average temperature eliciting a pain intensity of 50/100. This temperature will be used as the Heat Test Stimulus (see below).

#### Pre-CS Test Stimuli

Two Pre-CS Test Stimuli will be used in our study: a painful mechanical pressure and a painful heat stimulus. The Mechanical Test Stimulus will be administrated using the digital algometer, applied on the upper trapezius muscle at the mechanical pain threshold (see *Independent Variables—Mechanical pain*). The average pressure required to reach the mechanical pain threshold before and after the CS will be compared to assess CPM.

The second Test Stimulus will consist of a 2 min heat pain stimulation, delivered by the thermode applied on the left forearm of participants, at an individually tailored temperature selected to evoke pain levels of 50/100 (based on the pre-tests). Although the thermode temperature will be kept constant during the stimulation (following a gradual increase from 32°C to the target temperature, at a rate of 0.3°C/s), participants will be told that the temperature will randomly vary (increase, remain stable, or decrease). Participants will be asked to rate their pain during the stimulation using the CoVAS. This Heat Test Stimulus will serve two purposes: first, like the Mechanical Test Stimulus, it will be used as a pre- and post-CS comparator to assess CPM; second, it will be used to evaluate TS, which will be measured as the change in pain levels throughout the heat test stimulation.

#### Conditioning Stimulus (CS)

The conditioning stimulus, which serves to trigger CPM, will consist of the Cold Pressor Test, wherein participants immerse their right arm (up above the elbow) in a painfully cold water bath [10°C; (44)] for 2 min. This technique has been shown to consistently elicit CPM (92).

#### Post-CS Test Stimuli

The Heat Test Stimulus will be administered again immediately after the CS, using the same parameters as for the pre-CS stimulation. Following this, the Mechanical Test Stimulus will also be re-administered; again, four trials will be conducted and the average pressure corresponding to the pain threshold will be recorded. Comparisons between pre-CS and post-CS scores will be used to assess CPM.

## Ethical Aspects

This study will be conducted in accordance with the Helsinki Declaration and the International Council for Harmonization of Good Clinical Practices. Participants will be informed of the purpose and process of this study. We will collect their written informed consent, reminding them that participation in this study is purely voluntary and that they can withdraw at any time without negative repercussions, and we will preserve the confidentiality of their data. Participants will be asked to skip only the dose of their analgesic medication immediately preceding the experimental session, to minimize undue pain. The trial is registered at clinicaltrial.gov (NCT03376867).

## Statistical Considerations

### Sample Size

It is customary in predictive analysis to use a rule of thumb to calculate the sample size, wherein the number of subjects required for a multiple regression analysis is *n* = 10–15 subjects per variable (93). However, this rule often yields fitted models with limited predictive capabilities (94–98); as such, it is recommended that two cohorts be used: a discovery cohort, and a validation cohort—which, as their names suggest, are used to build the predictive model and to validate it, respectively (99).

We calculated a sample size of 360 participants for the discovery cohort, based on the smallest expected R^2^ (0.4) and the highest expected number of predictors (24) required to obtain a shrinkage coefficient (100) of at least 0.9 (101). The discovery cohort will be representative of the overall population, such that it will be comprised of 85% (*n* = 306) of healthy participants and 15% (*n* = 54) of patients suffering from chronic pain (102). The external validation cohort will be comprised of 54 (new) participants, corresponding to 15% of the size of the discovery cohort (103). We will use two such validation cohorts (*n* = 54 for both) in order to validate our model using both healthy participants and patients suffering from chronic pain. As such, a total of 468 participants will be recruited (360 healthy participants, and 108 patients suffering from chronic pain).

### TS and CPM Amplitude

The amplitude of TS (*A*_*TS*_) will be measured as the difference in pain intensity elicited by the thermode during the heat test stimulation (first instance the Heat Test Stimulus). We will subtract the pain level at 0 s (time the thermode reaches the target temperature) from the pain level at 120 s, so that a positive score represents an increase in pain level. The amplitude of CPM (*A*_*CPM*_) will be measured as the difference in pain level elicited by the thermode (calculated as the average of all pain scores taken throughout the 2 min test stimulation) before and after the cold water bath, and as the difference in pressure required to reach mechanical pain threshold before and after the cold water bath.

### Statistical Analyses

The sample will be characterized using descriptive statistics. The underlying assumptions of the regression model will be verified by analyzing the residuals distribution.

The main objective (identifying predictors of excitatory (TS) and inhibitory (CPM) endogenous pain mechanisms) will be achieved through the development of two predictive models (one for each mechanism). Development of a predictive model requires two steps: (1) Building and (2) Validation, which each use their own participant cohorts (see Sample Size section). Building involves a data-driven selection of the variables to be included as predictors in the model; for each variable, the beta coefficient is calculated and fitted to the model. Validation involves testing the model with data from new participants, in order to quantify the predictive capacity of the model. Validation will be conducted twice for each model: once with a healthy cohort, and once with a chronic pain cohort.

We will use a lasso regression on the discovery cohort to determine which variables will be included in the model for each mechanism. Lasso regression fits a linear model between an outcome and a set of predictors by adding a penalty term that shrinks coefficients associated to poor predictive capacity variables to 0 (i.e., Lasso automatically excludes poor predictors). This approach thus encourages simple and sparse models, and is widely used to perform variable selection in prediction contexts (103). The penalty term involves a coefficient (often denoted by lambda) that determines the amount of shrinkage to perform. This coefficient is often set so as to maximize a cross-validation estimate of the predictive capability of a model (103). Accordingly, we will select the shrinkage coefficient that minimizes the leave-one-out cross-validation estimate of the mean square prediction error. The resulting “optimal” shrinkage coefficient will be used within the lasso regression model, and the predictors and non-zero fitted parameters will be retained to constitute our predictive model.

In order to complete the validation (validation cohort), we will calculate the mean square prediction error of the predictive model for the healthy and chronic pain cohorts. If the mean square prediction error is smaller than 10%, the model will be considered to have a good predictive value. We will compare this predictive error between the two populations, to assess whether predictors are the same between healthy and patient populations.

The second objective (i.e., estimating reference TS and CPM values in healthy subjects) will use data from the 360 healthy participants (306 from the discovery cohort and the 54 from the healthy validation cohort). Participants will be separated into subgroups based on biological sex (male, female) and age (18–39, 40–59, and 60–79 years old), for a total of six subgroups. For each subgroup, a kernel estimation method will be used to obtain density curves, from which percentile reference charts will be generated (104). These analyses will be run separately for TS and CPM data.

## Discussion

To our knowledge, this is the first protocol that single handedly studies psychological, physiological and socio-demographic markers of endogenous pain modulation mechanisms.

The protocol we adopted to assess TS and CPM is frequently used in our laboratory; its reliability has been validated (10–12), and our expertise with the procedure and our familiarity with the equipment considerably reduces the risk of bias and confounding factors.

Chronic pain is a highly prevalent condition, for which there is currently no satisfying treatment approach. The success rates of surgery are subpar (105, 106); therapeutic exercises (107) only have limited success; and the current pharmacological approach, which relies mostly on drawn-out trial and error, is disheartening for both the patients, who struggle to see a finality to their pain, and the clinicians, who are confronted with their powerlessness in the face of their patients' everlasting ailment (108).

It is becoming more and more apparent that chronic pain treatment should focus not on the symptomatology—or type of condition—of chronic pain patients, but rather on the underlying imbalance in their pain modulation mechanisms. The amplitude of CPM and TS has been shown to correlate with the development of chronic pain conditions (109, 110), which imparts them with a predictive quality that could be extremely useful in clinical practice. Moreover, specific assessments of CPM and TS can effectively predict treatment success rates in patients suffering from chronic pain (27). As such, an individualized pharmacological treatment informed by the pain modulation profile of each patient could significantly improve treatment outcome. Unfortunately, current CPM and TS testing procedures remain too expensive and time-consuming to be used on a regular basis in hospitals or family medicine clinics.

This study will further our understanding of modulatory mechanisms and their relationship with a complex system of physiological, psychological and socio-demographic correlates, which will allow us to develop a predictive model to compute the pain modulation profile of individual patients based on their biopsychosocial characteristics. This model will consist in a simple equation in which clinicians can input the value of selected variables (obtained via simple clinical tests), in order to yield the predictive amplitude of their patients' CPM and/or TS response. The overarching goal of this study is to provide clinicians with a set of quick and cheap tests, easily applicable in day-to-day practice, in order to identify markers of pain modulation profiles and in turn orient pharmacological treatment, which will ultimately provide patients with a more hopeful outlook on their future and will lead to a more optimal use of ever limited health care resources. This protocol is the first step toward the development of a validated clinical tool.

## Data Availability Statement

The original contributions presented in the study are included in the article/supplementary material; further inquiries can be directed to the corresponding author/s.

## Ethics Statement

The studies involving human participants were reviewed and approved by the Comité d'éthique de la recherche du Centre intégré universitaire de santé et de services sociaux de l'Estrie - Centre hospitalier universitaire 3001, 12e Avenue Nord, Local HF-2929 Sherbrooke, QC J1H 5N4. The patients/participants provided their written informed consent to participate in this study.

## Author Contributions

MV wrote the manuscript with support from AC-L and MS. FCL helped the design statistical analysis. SM, GL, and LG conceived the study and helped draft the manuscript. All authors contributed to the article and approved the submitted version.

## Conflict of Interest

The authors declare that the research was conducted in the absence of any commercial or financial relationships that could be construed as a potential conflict of interest.
